# Diet self-management: A qualitative study of college students' experiences and perspectives

**DOI:** 10.3389/fpubh.2022.1059818

**Published:** 2022-12-12

**Authors:** Li Zhou, Yalin Chu, Lai Wei, Jing Wang, Xiaorong Zhu

**Affiliations:** Department of Health Management, School of Management, Zunyi Medical University, Zunyi, China

**Keywords:** college students, semi-structured interview, China, diet self-management, qualitative study

## Abstract

**Background:**

Overweight and obesity among college students have become an emergent public health concern, which may be effectively prevented by diet self-management (DSM). The purpose of this explorative study was to explore college students' experiences and perspectives on diet self-management (DSM), as well as its influencing factors.

**Method:**

Thirty-three college students were recruited from different universities in China. A qualitative method was used to conduct semi-structured interviews with audio recording to explore their DSM experience and factors that influence DSM. Data were analyzed using thematic analysis to develop themes related to DSM.

**Result:**

In the perception of what "good DSM" means, three themes and ten subthemes were identified: characteristics of good DSM (including 5 subthemes: regularity, balanced diet, no picky eating, good eating habits, and scientificity), method of good DSM (including three subthemes: self-control, adjusting, and making plans), and content of good DSM (including two subthemes: nutrition management and safety management). The influencing factors of DSM can be categorized into four levels of themes and 34 subthemes: individual, family, school, and social levels.

**Conclusion:**

The results of this qualitative research highlighted the complexity and multi-dimension of DSM and its influencing factors. Our findings may help to inform diverse and needs-based intervention approaches to improve DSM and promote healthy diet among college students so as to prevent overweight and obesity.

## Introduction

Globally, overweight and obesity have been addressed as leading public health concerns. Overweight and obesity are assessed by Body mass index (BMI), which is defined as a person's weight in kilograms divided by the square of a person's height in meters (kg/m^2^). The World Health Organization (WHO) defines overweight as BMI≥25 and obesity as BMI ≥30 (1). According to the most recent statistics released by the WHO, there are currently more than 1.9 billion overweight adults aged ≥18 years, accounting for 39% of the world's adult population (1). Of these overweight adults, over 650 million were obese, accounting for 13% of the world's adult population (1).

Although the worldwide prevalence of overweight and obesity has more than tripled in the general population since 1975 (1), the greatest increase in the prevalence of overweight and obese individuals has been concentrated in young adults aged 18–29 years (2). Young adults who attend college are at an even higher risk of overweight and obesity than non-college-attenders and have faster weight gain than the general population. According to the American College Health Association (3), the prevalence of overweight and obesity among college students has reached 34.1%, which is alarmingly high. College presents a time of transition from youth to adult, which is characterized by many key life changes such as moving away from home, starting tertiary education, and living with peers or partners (2). During this process of adaptation to the new environment, most college students are exposed to various risk factors such as fast-food consumption and sedentary behaviors that may predispose them to overweight and obesity (4).

College students who are overweight or obese are at increased risk of a series of non-communicable diseases, including cardiovascular diseases, diabetes, musculoskeletal disorders, sleep apnea, and even cancers (5–7). They also report a myriad of psychosocial problems including depression, anxiety, poor academic performance, stigmatization, peer victimization, and bullying (8, 9). Furthermore, overweight and obesity during college are likely to track into later adulthood, and their negative long-term health and psychosocial consequences may also accumulate with time (10). It is thus imperative to identify the risk factors for overweight and obesity in order to tailor prevention and treatment approaches among college students.

Although overweight and obesity are caused by interaction among multiple factors such as genetic, behavioral, and environmental factors (11), recent evidence suggests diet may be the most effective modifiable factor in preventing and reducing overweight and obesity (12, 13). Diet and nutrition have become a growing global health problem, as unhealthy diet has been dominating people's food consumption (14, 15). The past few decades have seen a global nutritional transition from a traditional, plant-based, and unrefined diet to an industrialized, energy-based, and refined diet (14, 15). Such a nutritional transition has led to the worldwide epidemic of obesity and subsequent non-communicable chronic diseases (NCDs), including cardiovascular diseases (CVD), digestive diseases, cancers, and mental illnesses (16–18). Dietary risk factors are among the most important contributors to mortality and morbidity from many NCDs in both developed and developing countries. In the United States, dietary risk factors have been estimated to account for ~26% of NCD deaths and 14% of disability-adjusted life-years (DALYs) (19). In Australia, nearly one-fifth (19.7%) of NCD deaths and 9.5% of NCD DALYs were attributable to dietary risk factors (20).

China has experienced unprecedented economic growth and urbanization since the economic reform and open-door policy in 1978 (21). The rapid socio-economic development in China has contributed to a remarkable nutrition transition from the traditional planted-oriented diet toward a high-energy Western diet dominated by high fat and high calories (22, 23). Such a nutrition transition has led to a substantially increased prevalence of obesity and subsequent NCDs (24). The latest national estimates for 2015–2019 showed a prevalence of 34.3% for overweight and 16.4% for obesity in adults aged ≥18 years (25). Similar increasing patterns were also shown in the prevalence of NCDs such as CVD, type-2 diabetes, and liver diseases (24). Of note, the nutrition transformation is especially obvious among Chinese college students, a major force in the rapid economic development (26). Chinese college students are faced with multiple life transitions and stressors, such as academic, employment, and debt pressure, and thus tend to engage in unhealthy eating behaviors (27). Furthermore, the convenient, cheap, and widely available take-out fast food also contributes to an obesogenic environment around universities, making college students especially susceptible to overweight and obesity (26).

Given abundant research has consistently shown unhealthy eating as a major contributor to obesity and related NCDs, self-management, which involves a series of daily activities or behaviors to maintain or improve health status (28), should be applied to healthy eating because it is applicable to chronic conditions as well as health promotion (29). Self-management emphasizes the pivotal role of the individual (30). However, compliance with such a seemingly simple practice is minimal (31, 32). Several lines of studies have proposed diet self-management as an essential driver of favorable diet behaviors that may help prevent obesity and NCDs (33, 34).

Diet self-management (DSM) refers to an individual's control of his or her eating behavior particularly to pursue a specific objective such as weight loss. It is a will to change one's unhealthy eating behaviors into a healthy diet and supports one's ambition, seriousness, and reliability (21). DSM represents an ideological shift that views individuals as active partners who are empowered to effectively manage their health by controlling their diet, instead of passively receiving advice and monitoring on diet control from their health providers or other people (35). Effective DSM requires the support provided to individuals and families to control their diet confidently and competently (35). From this perspective, people with DSM are competent to manage their eating behaviors without direct professional input from outside. Studies have consistently shown that DSM was associated with a healthy diet such as consumption of vegetables and fruits instead of high-fat and high-sugar food, which in turn will prevent overweight and obesity among college students (36–38).

The protective role of DSM in preventing overweight and obesity may be explained by Ajzen's Theory of Planned Behavior (39). According to this theory, behavior is determined by intention, which is further affected by attitudes toward the behavior, social or subjective norms, and perceived behavioral control (39). Students with better DSM hold more positive attitudes toward healthy eating behaviors and have higher self-control over their eating behaviors, which leads to a higher intention to adopt healthy eating. A higher intention will eventually translate into the behaviors of avoiding unhealthy food and adopting healthy eating and thus prevent overweight and obesity. On the contrary, those without DSM have no specific goals and requirements for their diet due to the lack of self-management consciousness, they are likely to engage in unhealthy eating behaviors such as binge eating, which may put them at high risk of overweight and obesity.

Although previous studies have shown the essential role DSM plays in informing healthy eating behaviors and preventing overweight and obesity among college students (36–38), they were mostly conducted in western counties. Little is known about DSM among college students in China, where the prevalence of overweight and obesity is rising sharply due to increased consumption of high-calorie foods and adoption of a more sedentary lifestyle (26, 40, 41). Furthermore, most previous studies on DSM among college students have used a quantitative study design that relies on questionnaires or survey instruments, which may not fully gain a deep and thorough understanding of college students' DSM and allows for exploration of a full range of responses as qualitative study design (42). In light of these limitations, we conducted the current study to explore perceptions of DSM among college students in China using a qualitative study design. To be specific, our research aimed to answer the following two research questions: (1) How do college students perceive DSM in China? (2) What factors influence their DSM? Our findings may provide useful and important guidance for future intervention programs to improve healthy eating behaviors among Chinese college students.

## Materials and methods

### Study design

Given the study aims, a qualitative descriptive research methodology was chosen to obtain a detailed understanding of college students' views of DSM. Qualitative semi-structured interviews were conducted one-on-one with college students. An interview guide with open-ended questions and probes was developed based on a review of the literature, and the interview guide was pilot-tested in a group of six college students. Based on the results from the pilot interviews, some minor modifications were made to better set up the interview. The final interview guide was divided into two themes: what “good DSM” means and what factors affected their DSM. Each interview began with opening questions about DSM in general, followed by more specific questions from the two themes in order to probe the participants for more information. For example, an opening question would be “Do you know anything about DSM?” and a more probing question would be “Can you list some examples of good DSM?” According to the different answers made by the participants, we further construct different follow-up questions during the interviews. The interview guide is shown in Supplementary File S1. All subjects gave their informed consent for inclusion before they participated in the study. Ethical approval was obtained from the Zunyi Medical University Medical Ethics Committee before the commencement of the study (Approval No. ZMUMEC-[2022]4-001, date of approval-29 March 2022).

### Sampling and recruitment

Inclusion criteria were college students residing in China, exclusion criteria were first-year college students because of their “limited” experience as college students. Purposive sampling was used to obtain a diverse sample of college students with maximum variation in key demographic factors in order to get as rich and diverse information as possible. Students from 5 different regions (East, North, West, South, and Central China), 26 different universities (such as Zhejiang University, Zunyi Medical University, and Qingdao University), 31 different majors (such as journalism, physics, and clinical medicine), and in 3 different school years (2–4 years) were recruited to ensure heterogeneity. The lead researcher (L.Z.) confirmed the eligibility and scheduled an interview. Before every interview, written consent and permission for audio recording were obtained from each participant. Each participant was reimbursed with a Chinese Renminbi of 30 Yuan as compensation for their time and participation. We recruited a final sample size of 33 college students for data saturation, which means no new findings or themes occurred from an analysis of newly collected data (43).

### Data collection

The semi-structured interviews were conducted online using Tencent QQ/WeChat by two researchers (L.Z. and Y.C.) who had experience in conducting qualitative interviews and did not have a relationship with the interviewees. Interviews were audiotaped with the permission of the participants and transcribed. Transcripts were translated into English and back-translated into Chinese for accuracy. The English-translated versions were then used for data analysis. All transcripts were not returned to participants for comment. Interviews ranged from 46 to 66 min with an average duration of 55 min.

### Data analysis

Data were managed using NVivo Version 12. Thematic analysis (44) was used to analyze the data. The audio-recorded interviews were transcribed verbatim and then coded. To ensure reliability and rigor, data analyses were carried out by two researchers (L.Z. and Y.C.). A deductive method was used for data analysis to develop themes around two predetermined areas in the interview guide: What is a good DSM? What are the influencing factors of DSM? First, transcripts were independently read by two team members (L.Z. and Y.C.) to identify preliminary codes. Second, one researcher (L.Z.) coded the data and clustered codes with similar meanings to form subthemes and themes. Another researcher (Y.C.) independently reviewed these codes, subthemes, and themes. Codes and themes were discussed and agreed upon between L.Z. and Y.C. A final set of themes and sub-themes that best represented the data set were formed, with counts made for each subtheme. Representative quotations were taken from the transcripts as example data to support the themes being derived from the content. The Consolidated Criteria for Reporting Qualitative Research guidelines, which covers the reporting of studies using interviews and focus groups, guided reporting of the study results (45).

## Results

### Sample details

Thirty-five college students from different universities in China expressed their interest in the study and were recruited. Two withdrew due to time constraints. The characteristics of students who participated in the interview were displayed in Supplementary File S2. The sample (*n* = 33) consisted of 13 male and 20 female students with a mean age of 20.9 (range: 19–23 years). Participants varied in major, school, and college years. In terms of the degree of DSM, most reported poor or no management at all (69.7%), and most expressed their worry about the effects of an unhealthy diet on health (75.7%).

### What “good diet self-management” means

In the perception of what “good diet self-management” means, three themes and ten subthemes were identified (Table 1): characteristics of good DSM (including 5 subthemes: regularity, balanced diet, no picky eating, good eating habits, and scientificity), method of good DSM (including 3 subthemes: self-control, adjusting, and making plans), and content of good DSM (including 2 subthemes: nutrition management and safety management).

**Table 1:** Summary of themes and subthemes generated of what “good diet self-management” means.

**Theme**	**Subtheme**	**Number**	**Representative quotes**
1. Characteristics of good DSM	1.1 Regularity	19	“Good DSM should be regular.” (S7)
			“In my opinion, the best diet self-management was to eat on time. For example, breakfast should be eaten before 9am. Breakfast and lunch should not be eaten at the same time and eating after 11 pm should be reduced.” (S22)
	1.2 Balanced diet	15	“Good diet management means that we should pay attention to the combination of meat and vegetables, the daily diet should include cereals, vegetables and fruits, beans, livestock, and poultry, etc.” (S15)
	1.3 No picky eating	2	“I think people with good DSM should firstly not be picky about food.” (S17)
	1.4 Good eating habits	2	“In my opinion, DSM was a behavior to promote one's health by maintaining reasonable and healthy eating habits, such as having three meals a day, eating more whole grains, fruits and vegetables, and reducing high-fat and high-sugar food.” (S13)
	1.5 Scientificity	2	“In my opinion, good DSM means that the amount, quality, elements, and nutritional structure of daily food intake meet certain scientific standards. You can refer to some scientific diet books to arrange your daily diet.” (S11)
2. Method of good DSM	2.1 Self-control	6	“Having some self-control over overeating, very sweet or spicy food.” (S11)
			“Good DSM is about learning to control your cravings for ‘delicious food.’ You can't just go after delicious food and let your body get hurt. Do not overeat, control weight on the basis of health, and not be only for the sake of beauty to pursue thin without considering healthy diet.” (S2)
	2.2 Adjusting	4	“In my opinion, good DSM is based on one's body weight. If someone is slightly overweight, he/she will reasonably control his/her diet so as to achieve a normal weight and vice versa.” (S27) “Eat and drink according to the weather.” (S18)
	2.3 Making plans	1	“Good diet management requires a clear plan and a list of food that is right for you.” (S26)
3. Content of good DSM	3.1 Nutrition management	27	“Know what to eat and how much to eat throughout the day.” (S14)
			“A good dietary self-manager will strictly control nutrients intake at a healthy level that is neither exceeding nor falling short.” (S14)
			“Good dietary management requires us to understand the nutritional content of different foods and pay attention to nutritional reasonable collocation.” (S25)
			“Follow the principle of having a good breakfast, a full lunch, and a light dinner.” (S18)
	3.2 Safety management	8	“Good DSM is to eat less high-cholesterol and non-fresh food, eat less high-oil and sugar food.” (S20)
			“Eat less junk food.” (S6)

#### Characteristics of good DSM

A key element of good DSM that was mentioned most frequently by participants was regularity, which included regularity in time and quantity. Participants believed good DSM means you need to intake an appropriate quantity of food at a fixed time every day to keep your body healthy. “*In my opinion, the best DSM was to eat on time. For example, breakfast should be eaten before 9 am. Breakfast and lunch should not be eaten at the same time, and eating after 11 pm should be reduced.” (S22)*.

Another characteristic of good DSM that was also mentioned frequently by participants was a balanced diet. According to the participants, a balanced diet means you need to intake a variety of healthy foods from different food groups to guarantee balanced nutrition. “*Good diet management means that we should pay attention to the combination of meat and vegetables, the daily diet should include cereals, vegetables and fruits, beans, livestock, and poultry, etc.” (S15)*.

In addition, participants also reported, “no picky eating”, “good eating habits”, and “scientificity” as the other three equally important characteristics of good DSM. They believed people with good DSM should not have preference over certain kinds of food and avoid other kinds of food. They should have a healthy lifestyle with good diet habits. They also highlighted the importance of following scientific guidance for food intake.

#### Method of good DSM

One major method of good DSM that has been most frequently mentioned was self-control. According to the participants, self-control is the ability to resist the temptation of unhealthy food such as those with high fat and high sugar but no nutrition. “*Having some self-control over overeating, very sweet or spicy food.” (S11)*.

Another way of good DSM was to adjust the diet according to physical or weather conditions, which was also frequently mentioned by the participants. “*In my opinion, good DSM is based on one's body weight. If someone is slightly overweight, he/she will reasonably control his/her diet so as to achieve a normal weight and vice versa.” (S27)*.

In addition, making plans has also been mentioned as a good DSM. One student explained that a good way of DSM should involve making a clear plan and choosing the correct food. “*Good diet management requires a clear plan and a list of food that is right for you.” (S26)*.

#### Content of good DSM

A major content of good DSM that has been most frequently mentioned by the participants was nutrition management. According to the participants, good DSM means strict control of food to ensure an appropriate level of nutrition. “*A good dietary self-manager will strictly control nutrients intake at a healthy level that is neither exceeding nor falling short.” (S14). “Good dietary management requires us to understand the nutritional content of different foods and pay attention to reasonable nutritional collocation.” (S25)*. In addition, participants also emphasized the importance of managing the nutrition of three meals a day. Specifically, one should not only have three meals a day but also have a different focus on the nutrition of each meal. “*Follow the principle of having a good breakfast, a full lunch, and a light dinner.” (S18)*.

Safety management was also identified as an important content of good DSM. Participants believed people with good DSM should avoid unhealthy food that will harm their health. “*Good DSM is to eat less high-cholesterol and non-fresh food, eat less high oil and sugar food.” (S6)*.

### Factors influencing DSM

When asked about what factors may affect their DSM, students listed a wide variety of influencing factors, which can be generally categorized into four levels of themes: individual, family, school, and social levels. Under each level, there are multiple subthemes, adding up to a total of 34 subthemes (Table 2).

**Table 2:** Summary of themes and subthemes generated on factors influencing DSM.

**Theme**	**Subtheme**	**Number of mentions**	**Representative quotes**
1. Individual-level factors	1.1 Knowledge of DSM	32	“Since I was only focused on learning and entertainment since childhood, I had a relatively shallow understanding of diet, and I didn't know much about how to match diet, so I didn't manage my diet.” (S25)
			“My knowledge of DSM was very limited. It was not until you asked me about it did I know that diet management included structure, nutrition, safety and so on.” (S33)
	1.2 Personal preferences	12	“I had no self-discipline and chose food according to my personal preference, so it was difficult for me to manage my diet.” (S13)
			“My taste preference would have a great influence on my diet management. I liked to eat sweet, flavorful things, so I ate more meat and protein. I did not like vegetables, so I ate fewer vegetables.” (S33)
	1.3 Self-management awareness	10	“Being aware of the importance of diet management helped me improve my diet management.” (S6)
			“Self-management awareness was very important in the face of all kinds of delicious food.” (S8)
	1.4 Self-discipline	10	“I didn't have enough self-control; people could not always operate in an established process like a machine. Today, I had my period, but I just wanted to eat sweet, icy, and spicy food, although they should be avoided, I wanted it so I ate it.” (S25)
	1.5 Mood	8	“Mood was also very important to me. Some people could eat well no matter what happened, but I did not want to eat when I was in a bad mood or in conflict or depressed or unhappy, which had a big impact on my diet.” (S1)
			“If I had a bad day, I didn't spend too much time on diet management and ate more casually.” (S15)
	1.6 Physical condition	7	“If my body was not feeling well or weak, it would definitely affect my diet. I would eat less or even skip meals.” (S1)
			“When my health condition was not good, I would strengthen diet management, I would also arrange my diet properly.” (S21)
	1.7 Laziness	4	“It was hard for me to manage my diet because I was lazy.” (S30)
	1.8 Weight management	4	“When I wanted to lose weight after overeating, I would choose to eat less, or even eat nothing, and pay special attention to the calories of food, so that I would forget the nutrition and the regular diet would be disrupted.” (S18)
	1.9 Dietary habit	4	“My eating habits predisposed me to certain foods and increased my cravings for those foods.” (S24)
	1.10 Economic factors	3	“Sometimes I wanted to eat nutritious food, but I had to pay more money, so I didn't buy it.” (S10)
	1.11 Thinking that young people's bodies are less prone to illness	2	“I don't think I am old enough to manage diet. Young people's bodies could suffer more. When my body was not healthy, I would learn how to manage it.” (S14)
	1.12 Lack of interest	1	“I don't want to waste time and energy in things that I was not interested in, even if it might affect my health.” (S25)
	1.13 Low dietary requirement	1	“My dietary requirements were too low.” (S20)
2. Family-level factors	2.1 Family supervision, reminder and education	9	“My parents told me to have meals on time since I was a child, which influenced me profoundly.” (S7)
			“When I was at home in summer and winter holidays, my parents were very strict with my diet, so I ate regularly.” (S8)
	2.2 Family eating habits	8	“When there were a large number of family members, it was difficult to fit one diet style to all family members. Family members vary in their perception and preference of diet, which may lead to variance in their actual diet management and thus cannot guarantee reasonable diet collocation.” (S2)
	2.3 Parents being in charge of the family diet	6	“The food was prepared by my parents, if there would be meals I didn't want to eat, diet nutrition would definitely be affected.” (S15)
	2.4 Special dietary needs of sick family members	4	“For example, my father's blood sugar was 6 points higher than the normal level, so he had to control his diet every day. Therefore, the meals made at home were targeted at diabetic patients, which affected my diet management.” (S12)
	2.5 Family diet knowledge	3	“Because my parents were farmers, the level of their education was not high, they didn't know much about diet.” (S25)
	2.6 Parents' demands to eat more	1	“If I was at home, my mother always asked me to eat more at any time.” (S18)
	2.7 Economic level of the family	1	“My family's economic level would affect DSM.” (S16)
3. School-level factors	3.1 The variety of meals in the canteen	9	“The abundance of food provided by the school had an impact on my diet management, which made me better adjust my diet structure.” (S9)
	3.2 Busy study	6	“Because I was busy with my studies and had not enough time to eat, I would not pay attention to balancing diet but only focus on the speed of eating.” (S15)
			“I usually stayed in the library for a whole day when it was near the end of the semester, I would not pay attention to diet, and usually prepared some bread, biscuits, and milk to go through the day.” (S27)
	3.3 School schedule	6	“I think the school schedule will affect my DSM, and an irregular schedule will disrupt my eating time. When there was no class, I would choose to stay in the dormitory for a day and could not eat on time. I sometimes ordered take-out in the middle of the day to get through the day.” (S2)
			“School had regular hours, so my meals were regular too.” (S26)
	3.4 The taste of the food prepared by the canteen	5	“When there were many things that tasted bad in the canteen, I would choose take-out food.” (S23)
	3.5 Peer influence	4	“My roommates could supervise each other, so each can form good DSM.” (S28)
			“If my classmates ate fried chicken at this meal, I could not help eating it at the next meal. After all, it was hard to resist the temptation of delicious food, then my diet management was broken.”(S22)
	3.6 Provision of a set meal	2	“If the school provided a set menu, I could order by myself.” (S32)
	3.7 Time spent waiting in line	2	“Sometimes the canteen on campus was so crowded that I didn't care too much about what I ate, as long as it was convenient.” (S19)
	3.8 Meal service hours in the canteen	1	“When meals were served longer, I would eat fewer instant noodles and choose the canteen more.”(S8)
	3.9 Price	1	“After the introduction of external catering companies in this semester, the price had obviously risen, and the stale food materials would make it difficult for me to choose.” (S8)
4. Social- level factors	4.1 Food culture and customs	12	“Food customs and local culture had influenced me from childhood to adulthood.” (S15)
			“In Guizhou, there was a custom of eating preserved meat and sausage in winter, but this kind of food was pickled, it was not very good for health, but I also ate it, so it had a great influence on me.” (S27)
	4.2 Food advertising	11	“Advertising could also affect me. If I saw an advertisement for a certain food on TV, good looks may also make me feel motivated to buy.” (S15)
			“Partying completely disrupted my eating habits and sometimes I may drink.” (S17)
	4.3 Social occasions and festivals	7	“New Year's Day or festival, the party with my relatives or friends would also affect my DSM, I could overeat.”(S9)
			“If an advertisement for a food continued to appear, my acceptance of that food would increase.” (S18)
	4.4 Food-related news reports	2	“When some news about some food safety investigation was disclosed, I would not choose it, I would choose the food with good evaluation.” (S24)
	4.5 Price of food	1	“The price of food also affected what I ate.” (S16)

#### Individual-level factors

Individual-level factors included 13 sub-themes: knowledge of DSM (*n* = 32), personal preferences (*n* = 12), self-management awareness (*n* = 10), self-discipline (*n* = 10), emotion (*n* = 8), physical condition (*n* = 7), laziness (*n* = 4), weight management (*n* = 4), dietary habit (*n* = 4), economic factors (*n* = 3), thinking that young people's bodies are less prone to illness (*n* = 2), lack of interest (*n* = 1), and low dietary requirement (*n* = 1).

Most participants mentioned the knowledge of DSM as an important influencing factor, with several participants reporting that their knowledge of DSM was limited, not thorough, or not comprehensive. “*My knowledge of DSM was very limited. It was not until you asked me about it did I know that diet management included structure, nutrition, safety, and so on.” (S33)*.

Participants expressed that personal food preferences also greatly influenced their DSM. They couldn't control their preferences, so they made food choices primarily based on preferences. “*I had no self-discipline and chose food according to my personal preference, so it was difficult for me to manage my diet.” (S13)*.

Self-management awareness was another frequently mentioned factor that affected how well they managed their diet. Participants believed that good self-management awareness could help them make healthy choices or improve their diet. “*Self-management awareness was very important in the face of all kinds of delicious food.” (S8)*.

Participants also reported good self-discipline as an important influencing factor of DSM. Good self-discipline means that you should have full control over the quantity, structure, and regularity of the food you intake, which affects how well you manage your diet. However, some participants expressed difficulty in maintaining good self-discipline. “*I didn't have enough self-control; people could not always operate in an established process like a machine. Today, I had my period, but I just wanted to eat sweet, icy, and spicy food, although they should be avoided, I wanted it so I ate it.” (S25)*.

Participants frequently reported that mood affected whether they would eat and how much they would eat. When they were under stress, they were more likely to engage in unhealthy eating behaviors to cope with stress, which affected their DSM. “*If I had a bad day, I didn't spend too much time on diet management and ate more casually.” (S15)*.

Participants revealed that physical condition was also an important factor that may affect their DSM. According to the participants, their health condition can affect their self-regulation of eating, either positively or negatively. “*If my body was not feeling well or weak, it would definitely affect my diet. I would eat less or even skip meals.” (S1)*.

In addition, some participants cited “laziness”, “weight management”, “dietary habits”, and “economic factors” as important influencing factors for DSM that may restrict their ability for diet management. Students reported that when they are lazy they could not manage their diet well. “*It was hard for me to manage my diet because I was lazy”. (S30)*. They also reported that concern about body weight made them neglect diet management. “*When I wanted to lose weight after overeating, I would choose to eat less, or even eat nothing, and pay special attention to the calories of food, so that I would forget the nutrition and the regular diet would be disrupted.” (S18)*. When participants stick to certain eating patterns for a long time, it will become difficult to change that pattern even though they knew it was not good. “*My eating habits predisposed me to certain foods and increased my cravings for those foods.” (S24)*. Economic factors also affected what and how much participants ate. “*If my parents gave me enough money for living expenses, I would eat whatever I wanted, order a variety of meals with better structure and nutrition.” (S8)*.

Other less frequently mentioned but equally important factors that may affect DSM included belief in illness resistance, lack of interest, and low dietary requirements. Two participants reported that young people's bodies were less prone to illness, so there was no need to manage diet and they delayed healthy choices until experiencing negative consequences. Some students viewed healthy eating as something that could be postponed. One participant reported a lack of interest in diet management as a reason for not managing it. Another participant reported low dietary requirements as a reason for not managing his diet.

#### Family-level factors

Family-level factors included 7 sub-themes: family supervision, reminder and education (*n* = 9), family eating habits (*n* = 8), parents being in charge of family diet (*n* = 6), special dietary needs of sick family members (*n* = 4), family diet knowledge (*n* = 3), parents' demands to eat more (*n* = 1), and economic level of the family (*n* = 1).

Family supervision, reminder, and education has been frequently reported as an important factor affecting DSM. Participants reported that family members' monitoring and suggestion of food intake would affect how well they manage their diet, and their home environment affected their eating behaviors. “*My parents told me to have meals on time since I was a child, which influenced me profoundly.” (S7)*. Participants with loose family supervision on eating habits were likely to have poor management of their diet. “*They always told me to pay attention to the quantity and quality of diet, but they just told me orally without putting pressure, so I didn't pay much attention to diet management.” (S25)*.

Family eating habits were also frequently mentioned by participants as affecting their DSM. In most families, moms are responsible for preparing meals and thus have a major impact on children's eating habits. “*Influenced by my mother, I usually paid attention to food collocation and balancing meat and vegetables. I would specially prepare vegetarian and meals, usually eat a little fruit after dinner.” (S26)*. However, participants also complained about the difficulty in satisfying the diverse diet needs of various family members, especially for a big family, making it hard to maintain good DSM. “*When there were a large number of family members, it was difficult to fit one diet style to all family members. Family members vary in their perception and preference of diet, which may lead to variance in their actual diet management and thus cannot guarantee reasonable diet collocation.” (S2)*.

Participants emphasized the important role parents played in forming their eating habits when they were in charge of the family diet. For most families, parents were responsible for preparing food and cooking meals, which would affect participants' diet management. “*The food was prepared by my parents, if there would be meals I didn't want to eat, diet nutrition would definitely be affected.” (S15)*.

Participants revealed that the special dietary needs of sick family members may also affect their DSM. “*For example, my father's blood sugar was 6 points higher than the normal level, so he had to control his diet every day. Therefore, the meals made at home were targeted at diabetic patients, which affected my diet management.” (S12)*.

In addition, family diet knowledge, parents' demands to eat more, and family economic level may also have an impact on participants' DSM. Participants from rural areas mentioned the lack of health literacy in their families led to their poor diet management. One participant complained about the pressure of eating more from his mom, which affected his DSM. Another participant emphasized the importance of family income in determining their diet management.

#### School-level factors

School-level factors included 9 sub-themes: the variety of meals in the canteen (*n* = 9), busy study (*n* = 6), school schedule (*n* = 6), the taste of the food prepared by the canteen (*n* = 5), peer influence (*n* = 4), provision of a set meal (*n* = 2), time spent waiting in line (*n* = 2), meal service hours in the canteen (*n* = 1), and price (*n* = 1).

The variety of meals in the canteen has been frequently cited as an important factor that influenced participants' DSM. Students may adapt their diet habits according to food types in the school canteen. “*The abundance of food provided by the school had an impact on my diet management, which made me better adjust my diet structure.” (S9)*. Participants also complained of limited food choices due to the lack of food diversity in the school canteen, which affected their DSM. “*School would affect my DSM because the food in school was not diverse enough, which limited my choice of food”. (S31)*.

The busy study has also been frequently mentioned as an important influencing factor of DSM. College students are constantly challenged by academic responsibilities and may prioritize study over healthy food choices. Participants reported that a busy study would disturb their DSM, they only paid attention to the speed of eating without considering a healthy diet. “*Because I was busy with my studies and had not enough time to eat, I would not pay attention to balancing diet but only focus on the speed of eating.” (S15)*.

Participants stressed the importance of school schedules in affecting DSM. According to the participants, a regular school schedule means you have to come to classes at a fixed time and have meals before and after classes, which makes them stick to regular diet habits. “*School had regular hours, so my meals were regular too.” (S26)*.

Participants reported that the taste of the food prepared by the canteen would affect their choices, such as whether to eat in the canteen or what to eat. Participants were more likely to eat take-out food that was usually high in calories when they were not satisfied with the taste of food from school, which may lead to poor DSM. “*When there were many things that tasted bad in the canteen, I would choose take-out food.” (S23)*.

Peer influence was also an important factor that couldn't be ignored, such as modeling behavior, encouragement, and peer support. Peers' good eating habits may help participants form good DSM, while bad eating habits of peers may lead to poor diet management of the participants. “*My roommates could supervise each other, so each can form a good DSM.” (S28)*.

In addition, participants mentioned their DSM was also affected by “provision of a set meal”, “waiting in line”, “service hours in the canteen”, and “price”. Participants expressed a high willness to have a set menu, which will facilitate better DSM. Participants also complained of the crowded waiting line at the school canteen made them prioritize convenience over health and led to poor diet management. When there was a long waiting line in the school canteen, participants may focus only on convenience and speed in order to save time. “*Sometimes the canteen on campus was so crowded that I didn't care too much about what I ate, as long as it was convenient.” (S19)*. One participant reported that meal service hours in the canteen would make them more willing to eat in the canteen. Another participant reported that the reasonableness of the price will affect DSM.

#### Social-level factors

Social-level factors included 5 sub-themes: food culture and customs (*n* = 12), food advertising (*n* = 11), social occasions and festivals (*n* = 7), food-related news reports (*n* = 2), and price of food (*n* = 1).

Participants reported that food culture and customs would affect DSM. Culture has long been recognized as a key factor that profoundly affected people's dietary decisions. “*Food customs and local culture had influenced me from childhood to adulthood” (S15)*. In addition, food culture varies according to various regions, and people from certain regions may have some special diet habits and preferences for food, which may affect their DSM. “*In Guizhou, there was a custom of eating preserved meat and sausage in winter, but this kind of food was pickled, it was not very good for health, but I also ate it, so it had a great influence on me.” (S27)*.

Food advertising was also frequently mentioned as an important influencing factor that would affect DSM. Food advertisements, due to their repeated appearance and good visual effects, would arouse their curiosity and increase their acceptance of the food. “*If an advertisement for a food continued to appear, my acceptance of that food would increase.” (S18)*.

Participants reported that their DSM may also be affected by social occasions and festivals and mostly in a negative way. When family and friends get together, they are more likely to engage in overeating and overdrinking behaviors, which may disrupt their usual normal diet management plan such that regularity, safety, and quantity would be ignored. “*Partying completely disrupted my eating habits and sometimes I may drink.” (S17)*.

In addition, food-related news reports and prices would also affect participants' DSM and choice of food. Disclosure of food problems may lead to participants' avoidance of certain food and change in their dietary choices. “*When some news about some food safety investigation was disclosed, I would not choose it, I would choose the food with good evaluation.” (S24)*. One participant reported that the price of food would affect DSM.

## Discussion

### Summary of the findings

The purpose of this explorative study was to understand the perception of DSM and what constitutes good DSM among college students in China. Furthermore, we aimed to identify influencing factors that may affect college students' DSM. In the perception of what “good DSM” means, three themes and ten subthemes were identified: characteristics of good DSM (including 5 subthemes: regularity, balanced diet, no picky eating, good eating habits, and scientificity), method of good DSM (including 3 subthemes: self-control, adjusting, and making plans), and content of good DSM (including 2 subthemes: nutrition management and safety management). When asked about the influencing factors of DSM, participants identified a wide range of various factors that may affect DSM, which can be categorized into four levels: individual level (13 subthemes: knowledge, personal preferences, self-management awareness, self-discipline, emotion, etc.), family level (7 subthemes: family supervision, reminder, and education, family eating habits, parents being in charge of family diet, etc.), school level (9 sub-themes: the variety of meals in the canteen, busy study, school schedule, etc.), and social level (5 sub-themes: food culture and customs, food advertising, social occasions, and festivals etc.) Our findings provide comprehensive and in-depth information on college students' perception of DSM and help us better understand their knowledge, attitudes, and behaviors regarding diet. In addition, we identified multiple influencing factors of DSM across the individual, family, school, and social levels. Our results provide important guidance for future intervention programs to improve college students' DSM by taking comprehensive and targeted measures. Improvement of DSM will help college students to engage in and sustain healthy eating behaviors, which will eventually help them to prevent overweight and obesity.

### What is good DSM

In terms of what is good DSM, participants were able to identify various elements of good DSM that cover the characteristics, method, and content of DSM. In a word, good diet management requires individuals to use a variety of effective management methods, to pay attention to the management of nutrition and safety. In addition, good DSM has unique characteristics that distinguish itself from bad management, which will help us understand the concept better and provide guidance to implement good DSM. DSM is a complex concept that involves multiple aspects, and our findings showed that college students had some understanding of the multidimensionality of good DSM. A core element underlying the conceptualization of DSM is self-efficacy, defined as an individual's belief in his or her ability to execute behaviors necessary to produce positive health outcomes (46). Self-efficacy was deeply rooted in the various aspects of DSM including characteristics (regularity, scientificity, balance, etc.), methods (control, plan, and adjustment), and content (nutrition management and safety management). The construct of DSM was in line with Bandura's Self-Efficacy Theory (SET), which focuses on self-activation and its implications (46–48). According to the SET (46–48), people with good DSM have good self-judgment of their ability to manage their diet (SE expectation) as well as the positive health outcomes following their successful diet management (outcome expectation). For the characteristics of good DSM, participants understood that people with good DSM should develop good eating habits and have a regular and balanced diet, such as having three meals a day and consuming diverse food with various nutrition. They should follow scientific guidelines when choosing what to eat and not be picky about food. Regarding the method of good DSM, participants knew how to take measures to achieve good DSM and stressed the importance of making plans, adjusting diet according to needs, as well as having discipline over what to eat and what to avoid. As for the content of good DSM, participants highlighted management of nutrition and safety as important elements of good DSM, the ultimate goal of good DSM is a healthy diet, and nutrition and safety are two important aspects of a healthy diet (49). A healthy diet requires not only nutrition management to select food that is good for the body, but also safety management to avoid unhealthy food that is harmful to your health, such as sugar-sweetened beverages, fast food, or sweet or salty snacks (50, 51). All these results suggest that college students had a comprehensive understanding of the multidimensional concept of good DSM. Correct knowledge and cognition are the premise of good behavior, which will facilitate favorable diet behaviors and prevent overweight and obesity.

### Influencing factors of DSM

As for the influencing factors of DSM, four levels of themes have been identified, including individual, family, school, and social factors. These findings align with Bandura's Social Cognitive Theory (SCT), the most widely used model to explain health-related motivations and behaviors (52). SCT proposes that health-associated behaviors were developed through cognitive processes based on a three-way mutual interaction between person, environment (physical and social), and behavior, referred to as triadic reciprocal causation (52–54). According to SCT, DSM was affected by the interaction of several constructs from different levels, such as knowledge and awareness from the individual level, supervision, and reminder from the family level, school schedule and peer pressure from the school level, and culture and festivals from the social level. Therefore, the SCT provides opportunities to examine and explore the interaction between personal and environmental factors that influence DSM.

Individual level factors included a wide range of personal factors such as knowledge, awareness, preferences, self-discipline, etc., which were consistent with previous studies. For instance, in Deliens et al.'s (55) qualitative study on college students' eating behavior, students reported being influenced by individual factors such as taste preferences, self-discipline, time, and convenience. Cluskey et al.'s (56) study cited lack of knowledge and awareness as major barriers for college students to make healthy diet choices and adapt to college life healthily. Our findings provide useful implications for future interventions to improve DSM by targeting multiple personal factors. As evidenced by Kelly et al.'s (57) systematic review of the effectiveness of dietary interventions among college students, interventions on the individual levels showed promise in improving students' DSM and dietary behaviors. More importantly, the inclusion of self-regulation components such as self-monitoring and goal setting may maximize intervention outcomes (57). This suggests that schools could carry out some intervention programs aimed at strengthening students' self-regulation and self-discipline skills on diet, which can help students to maintain good DSM and prevent overweight and obesity (58). In general, our findings added further support for the importance of targeting personal factors in improving DSM and diet behaviors among college students.

Family level factors included several factors such as knowledge, supervision, reminder, special needs, and economy. These findings suggest that family also plays an essential role in helping college students to form good DSM and are in line with the Individual and Family Self-Management Theory (IFSMT) (29). According to IFSMT, DSM is a process in which individuals and families influence each other to achieve a healthy diet by using knowledge, beliefs, self-regulation, monitoring, reminder, etc. (29). The individual or family assumes responsibility for their eating behaviors and incorporating healthy eating into their daily functioning. Family environment, including parents' dietary knowledge, reminder, monitoring, and eating habits has a huge impact on children's diet behavior (59–61). Parents serve as models for eating behavior and transmit dietary attitudes throughout the upbringing of their offspring (59, 60). The dietary routines, eating beliefs and behaviors learned from the family tend to affect children throughout adulthood (59, 60). These findings suggest that family members' active participation in college students' healthy diet management should be encouraged and promoted to improve college students' DSM and healthy eating behaviors.

School-level factors such as meals provided by the school canteen, school schedules, and peer pressure also affected college students' DSM, which was consistent with previous studies (55, 62, 63). As a major place where college students spent most of their time studying and living, school plays an important role in helping students form good DSM. Schools should create a healthy food environment, which meets the requirements of the Health Promoting Schools (HPSs) framework developed by WHO (64), such as enriching the variety of meals as well as improving the taste of meals and so on. In addition, considering the busy study schedule of college students, the school should also take appropriate measures to reduce students' waiting time for meals, which may be realized by flexible and longer opening hours of canteens. Furthermore, college students' DSM may be greatly affected by their peers' knowledge, attitudes, encouragement, and behaviors (65). Peers may serve as a negative role model in normalizing stress-related eating behaviors (65), indicating that peer education and intervention are needed to help college students form good and healthy DSM.

Similar to previous studies (63, 66), our study showed that social-level factors such as culture, advertising, festivals, and news also affected college students' DSM. The important role of social factors in affecting diet and obesity has been well documented in the literature. In a cross-cultural analysis of prevention models to fight obesity in various countries, Gracia-Arnaiz et al. (67) found that sociocultural factors played a more important role than biological and/or behavioral factors in informing healthy diet behaviors and preventing obesity. It is thus suggested that diet and obesity, as a multidimensional public health problem, should be dealt with in a holistic, open, and cross-disciplinary manner to ensure a comprehensive understanding (68). Dietary culture plays an important role in college students' dietary thinking and behaviors (69). For example, the traditional Chinese diet is very healthy with low calories, but some young people tend to engage in unhealthy eating habits under the influence of Western diet culture (70, 71). Therefore, in addition to building and maintaining healthy food culture, we should also resist unhealthy diet culture to provide a healthy cultural environment for diet management. In addition, studies have shown that television (TV) advertisements for food may increase people's preferences and overconsumption of food, leading to overweight and obesity (72, 73). It is thus suggested that food advertising should be health-oriented and advertising for unhealthy diets should be limited to prevent misleading college students to form bad DSM. Although festivals and social events do not happen every day, they have the potential to affect college students' eating attitudes and behaviors. Therefore, more public education and propaganda should be provided to form an appropriate and healthy diet during festivals and social events.

### Strengths and limitations

There are several strengths to this study. First, this is the first study to explore and report on college students' DSM in China, which fills in the research gap and provides valuable insights into this topic to inform the development of intervention strategy. Second, the qualitative methodology allowed for the generation of in-depth and rich data to gain a deep and thorough understanding of college students' experiences and perspectives on DSM. Some limitations of this study should also be noted. First, actual diet behaviors were not assessed in this study, we cannot link college students' eating behaviors to their perceptions of good DSM, which warrants further research to establish such associations in the future. Second, selection bias may exist since the sample was recruited from certain universities and participants joined the study voluntarily. The findings from this sample may not represent the experiences and perspectives of other students not sampled in this study. Third, all interviews were conducted online instead of face-to-face, which may affect the quality of data collection due to the lack of efficient real-person interaction.

## Conclusion

This study provides important insights into the perceptions of college students' DSM and the influencing factors of their DSM. Our results showed that college students were able to identify various characteristics, methods, and contents of good DSM, indicating their basic understanding of the complex and multi-dimensional concept of DSM. In addition, college students' DSM was shaped by the interaction of multiple factors that covered individual, family, school, and social levels, suggesting that multi-level, diverse, and comprehensive intervention approaches are needed to improve DSM among college students. Results from this study identify potential areas of intervention, such as improving students' self-regulation and self-discipline skills (individual level), promoting family members' role model behaviors (family level), extending school canteens' opening hours (school level), and limiting advertisement for unhealthy diets (social level). Future research is warranted to identify the most cost-effective line of interventions to improve DSM among college students.

## Data availability statement

The original contributions presented in the study are included in the article/Supplementary material, further inquiries can be directed to the corresponding author/s.

## Ethics statement

The studies involving human participants were reviewed and approved by Zunyi Medical University Medical Ethics Committee. The patients/participants provided their written informed consent to participate in this study.

## Author contributions

LZ obtained funding and designed and led the implementation of the study. LZ and YC wrote the first draft, analyzed the data, and conducted the interviews. LZ, YC, and LW developed the interview guides. LZ, YC, LW, and JW contributed to interpretation of the results. LZ, YC, JW, and XZ revised the manuscript. All authors have read and agreed to the published version of the manuscript.

## Supplementary material

Supplementary File S1 Interview guide for college students' diet self-management.

Supplementary File S2 Baseline characteristics of study participants.
