# Supplementary material for: Diet self-management: A qualitative study of college students' experiences and perspectives
Source: Front Public Health. 2022 Dec 12;10:1059818. doi: 10.3389/fpubh.2022.1059818 (PMC9790922; doi:10.3389/fpubh.2022.1059818)
Supplement: Supplementary File S1 — Interview guide for college students' diet self-management. [file Table_1.DOCX]

[Interview](javascript:;) guide for college students’ diet self-management

Please give me some your basic information：school, major, age, gender, year in college, and hometown.

1. Let's talk about your diet.

Do you think your diet is healthy？What are the problems with your diet? Do you know what diseases an unhealthy diet can cause? Do you ever worry about your health if you have an unhealthy diet?

1. Let's talk about the diet self-management (DSM).

Do you know anything about DSM? （If the respondent says yes, we will ask the participant to specify what the DSM is to provide the basis for subsequent interviews, if the respondents do not know what DSM is, we will explain the definition of DSM: DSM is to achieve the goal of healthy eating through the control of the individual's own diet)

Do you think what is good diet self-management？(According to the answers of the respondents, the interviewers will supplement the answers, so that the respondents have a complete understanding of good diet self-management and lay the foundation for the subsequent interview. )

Do you practice diet self-management? Do you think you manage your diet well？What are the factors that promote and/or hinder your diet self-management？（The interviewer suggested that the interviewees could answer from the internal (self) and external (family, school, society, etc.) factors. ）

Thanks a lot.
