# Supplementary material for: Diet self-management: A qualitative study of college students' experiences and perspectives
Source: Front Public Health. 2022 Dec 12;10:1059818. doi: 10.3389/fpubh.2022.1059818 (PMC9790922; doi:10.3389/fpubh.2022.1059818)
Supplement: Supplementary File S2 — Baseline characteristics of study participants. [file Table_2.DOCX]

Table S1 Baseline characteristics of study participants.

| **N0** | **School** | **Major** | **Age** | **Gender** | **Year in college** | **Hometown** | **The degree of DSM** | **Whether to worry about the effects of unhealthy diet on health** |
| --- | --- | --- | --- | --- | --- | --- | --- | --- |
| S1 | Guizhou Normal University | Astronomy | 22 | M | 4th | Rural areas | Good | No |
| S2 | Guizhou Normal University | Physics | 23 | F | 4th | Rural areas | Good | Yes |
| S3 | Zhengzhou University | Preventive medicine | 23 | F | 4th | Rural areas | Bad | Yes |
| S4 | Hunan University of Chinese Medicine | Medical radiology | 23 | F | 4th | Rural areas | Bad | No |
| S5 | Yanshan University | Software engineering | 20 | M | 3rd | Urban areas | Bad | Yes |
| S6 | Liaoning University | Applied chemistry | 20 | F | 2nd | Urban areas | No | Yes |
| S7 | Zunyi Medical University | Public management | 22 | F | 4th | Rural areas | Bad | No |
| S8 | Northeast Forestry University | Economic statistics | 20 | F | 3rd | Urban areas | Bad | Yes |
| S9 | Northeastern University | Artificial intelligence | 21 | M | 3rd | Urban areas | Bad | Yes |
| S10 | Nanjing University | Industrial economics | 20 | M | 3rd | Urban areas | Bad | Yes |
| S11 | Zunyi Medical University | Psychologic medicine | 22 | F | 3rd | Rural areas | Bad | No |
| S12 | Zunyi Medical University | Social sports guidance and management | 22 | M | 3rd | Rural areas | Good | Yes |
| S13 | Zunyi Medical University | Public management | 23 | F | 4th | Rural areas | No | Yes |
| S14 | Jiujiang University | International economics and trade | 20 | M | 2nd | Rural areas | Bad | Yes |
| S15 | Harbin Institute of Information Technology | E-commerce | 20 | M | 2nd | Rural areas | Good | No |
| S16 | Jiangxi Agricultural University | Business administration | 20 | M | 2nd | Rural areas | No | Yes |
| S17 | Liaoning University | Journalism | 20 | F | 3rd | Urban areas | No | Yes |
| S18 | Shanxi University of Chinese Medicine | Marketing | 21 | F | 4th | Rural areas | Bad | Yes |
| S19 | China University of Petroleum（East China） | Environmental protection equipment engineering | 20 | F | 2nd | Rural areas | Good | No |
| S20 | Guangdong University of Education | Ideological and political education | 19 | F | 2nd | Urban areas | No | Yes |
| S21 | Zunyi Medical University | Public management | 20 | F | 2nd | Rural areas | Good | Yes |
| S22 | North China University of Technology | Accountancy | 21 | F | 3rd | Rural areas | Good | Yes |
| S23 | Qiongtai Normal University | Chinese language and literature | 21 | F | 3rd | Rural areas | Good | No |
| S24 | Hunan Institute of Technology | Logistics management | 21 | M | 4th | Rural areas | Bad | Yes |
| S25 | Zhejiang University | Information technology | 20 | M | 2nd | Rural areas | No | Yes |
| S26 | Qingdao University | Management | 20 | M | 2nd | Rural areas | Good | No |
| S27 | Guizhou Medical University | Clinical medicine | 22 | F | 3rd | Rural areas | Good | Yes |
| S28 | Nankai University | Business and economics | 21 | F | 2nd | Rural areas | Bad | Yes |

Table S1. Cont.

| **N0** | **School** | **Major** | **Age** | **Gender** | **Year in college** | **Hometown** | **The degree of DSM** | **Whether to worry about the effects of unhealthy diet on health** |
| --- | --- | --- | --- | --- | --- | --- | --- | --- |
| S29 | Chongqing Normal University | English major | 19 | F | 2nd | Rural areas | Bad | Yes |
| S30 | Hainan Tropical Ocean University | Horticulture | 22 | F | 4th | Rural areas | No | Yes |
| S31 | Guizhou Medical University | Nursing | 23 | F | 4th | Rural areas | Bad | Yes |
| S32 | Tianjin College, University of Science and Technology Beijing | Financial engineering | 20 | M | 2nd | Rural areas | No | Yes |
| S33 | Shanxi Agricultural University | Traditional Chinese medicine | 20 | M | 2nd | Rural areas | Bad | Yes |
